# Supplementary material for: Diversity of Volatile Compounds in Ten Varieties of Zingiberaceae
Source: Molecules. 2022 Jan 17;27(2):565. doi: 10.3390/molecules27020565 (PMC8777948; doi:10.3390/molecules27020565)
Supplement: Supplementary file 1 [file molecules-27-00565-s001.zip › molecules-1533005-supplementary.pdf]

Supplementary Table S1. The qualitative and quantitative analysis of compounds of ten plants based on GC-MS

**Number**	**RT (min)**	**Compound name**	**CAS Number**	***Curcuma rubescens* Roxb.**	***Curcuma attenuata* Wall.**	***Curcuma aeruginosa* Roxb.**	***Costus comosus* var. *bakeri* (K.Schum.) Maas**	***Zingiber zerumbet* (L.) Smith**	**Hongfengshou**	***Alpinia oxyphylla* Miq.**	***Alpinia pumila* Hook.F.**	***Hedychium brevicaule* D.Fang**	***Hedychium coronarium* Koeng**
1	5.82	α-pinene	80-56-8	0.16	nd	nd	nd	nd	nd	nd	nd	nd	nd
2	5.85	cyclofenchene	488-97-1	nd	nd	33.83	nd	2.23	nd	25.47	1.52	16.21	1.13
3	6.16	camphene	79-92-5	nd	nd	nd	18.11	nd	nd	nd	2.00	nd	nd
4	6.72	sabinene	3387-41-5	nd	nd	nd	6.72	nd	nd	nd	nd	nd	3.11
5	6.77	L-β-pinene	18172-67-3	nd	nd	65.85	nd	nd	nd	32.06	nd	29.81	nd
6	6.77	β-pinene	127-91-3	0.60	nd	nd	nd	6.18	nd	nd	8.32	nd	nd
7	7.36	α-phellandrene	99-83-2	nd	nd	nd	nd	3.52	nd	nd	nd	nd	nd
8	7.84	m-cymene	535-77-3	nd	nd	nd	1.60	nd	nd	nd	0.21	nd	nd
9	7.84	cis-chrysanthenyl formate	241123-18-2	nd	nd	nd	nd	2.70	nd	nd	nd	nd	nd
10	7.97	trans-sabinene hydrate	17699-16-0	nd	nd	nd	nd	nd	nd	32.06	2.47	0.39	nd
11	7.99	eucalyptol	470-82-6	nd	nd	nd	nd	0.30	nd	0.75	nd	1.21	3.43
12	9.57	linalool	78-70-6	nd	nd	nd	nd	0.41	nd	nd	1.34	nd	nd
13	10.25	2,4,6-trimethyl-1,3,6-heptatriene	24648-33-7	nd	nd	nd	nd	nd	nd	0.52	nd	0.45	nd
14	10.56	L-trans-pinocarveol	547-61-5	nd	nd	nd	nd	nd	nd	nd	0.20	nd	nd
15	10.71	(+)-2-bornanone	464-49-3	nd	nd	nd	nd	nd	nd	nd	5.60	nd	nd
16	11.02	isoborneol	124-76-5	nd	nd	nd	nd	nd	nd	nd	0.13	nd	nd
17	11.15	pinocarvone	30460-92-5	nd	nd	nd	nd	nd	nd	nd	0.17	nd	nd
18	11.22	endo-borneo	507-70-0	nd	nd	nd	9.97	nd	nd	nd	0.11	nd	nd
19	11.22	α-campholenal	4501-58-0	nd	nd	nd	nd	1.24	nd	nd	nd	nd	nd
20	11.70	cryptone	500-02-7	nd	nd	nd	nd	nd	2.24	nd	0.53	nd	nd
21	11.78	α-terpineol	98-55-5	nd	nd	nd	nd	0.35	nd	nd	nd	nd	nd
22	11.93	(-)-myrtenol	19894-97-4	nd	nd	1.67	nd	nd	nd	0.31	0.64	0.44	nd

23	11.95	myrtenal	564-94-3	nd	nd	nd	nd	21.42	nd	nd	nd	nd	nd
24	12.94	2-methyl-3-phenyl-propanal	1000131-87-6	nd	nd	nd	nd	8.43	nd	nd	nd	nd	nd
25	13.38	α-thujenal	57129-54-1	nd	nd	nd	nd	1.67	nd	nd	nd	nd	nd
26	13.59	α-citral	141-27-5	nd	nd	nd	nd	2.80	nd	nd	nd	nd	nd
27	13.74	perillaldehyde	2111-75-3	nd	nd	nd	nd	0.84	nd	nd	nd	nd	nd
28	14.00	L-bornyl acetate	5655-61-8	nd	nd	nd	20.72	nd	nd	nd	nd	nd	nd
29	14.13	1,7,7-trimethylbicyclo[2.2.1]hept-5-en-2-one	22516-10-5	nd	3.47	nd	nd	nd	6.40	nd	nd	nd	nd
30	15.06	5-isopropenyl-2-methyl-7-oxabicyclo[4.1.0]heptan-2-ol	1000185-00-9	nd	nd	nd	nd	nd	1.97	nd	nd	nd	nd
31	15.17	elemene isomer	1000414-93-6	nd	nd	nd	1.16	nd	nd	nd	nd	nd	nd
32	15.17	δ-elemene	20307-84-0	nd	nd	nd	nd	nd	nd	nd	nd	nd	3.77
33	16.04	copaene	3856-25-5	nd	nd	nd	nd	0.28	nd	nd	nd	nd	nd
34	16.37	β-elemene	515-13-9	nd	nd	nd	nd	0.45	nd	nd	nd	nd	6.60
35	16.83	4-hydroxy-6-isopropyl-3-methylcyclohex-2-enone	55955-53-8	nd	nd	nd	nd	nd	1.52	nd	nd	nd	nd
36	16.96	α-santalene	512-61-8	nd	nd	nd	nd	nd	nd	nd	0.49	nd	nd
37	17.01	caryophyllene	87-44-5	nd	nd	nd	nd	nd	nd	0.31	0.42	4.17	2.99
38	17.12	(Z)-β-curcumen-12-ol	698365-10-5	nd	nd	nd	nd	nd	nd	nd	nd	nd	3.25
39	17.20	β-copaene	18252-44-3	nd	nd	nd	nd	nd	nd	nd	nd	nd	1.09
40	17.25	β-germacrene	15423-57-1	nd	nd	nd	nd	nd	nd	nd	nd	nd	8.99
41	17.27	cis-α-bergamotene	18252-46-5	nd	nd	nd	nd	nd	nd	nd	0.14	nd	nd
42	17.65	cis-β-farnesene	28973-97-9	nd	nd	nd	nd	nd	nd	0.40	nd	0.41	nd
43	17.72	humulene	6753-98-6	nd	nd	nd	nd	nd	nd	0.89	0.26	0.84	nd
44	18.14	4,11-selinadiene	1000192-43-5	nd	nd	nd	nd	nd	nd	nd	0.28	nd	nd
45	18.24	α-curcumene	644-30-4	nd	nd	nd	nd	nd	nd	nd	nd	nd	1.06
46	18.35	aristolochene	26620-71-3	nd	nd	nd	1.49	nd	nd	nd	nd	nd	nd

47	18.40	β-selinene	17066-67-0	nd	nd	nd	nd	nd	nd	nd	0.38	nd	nd
48	18.49	α-cedrene	469-61-4	nd	nd	nd	nd	0.49	nd	nd	nd	nd	nd
49	18.57	curzerene	17910-09-7	nd	nd	nd	nd	nd	nd	nd	nd	nd	3.89
50	18.71	α-farnesene	502-61-4	nd	nd	nd	nd	nd	nd	nd	nd	nd	2.54
51	18.76	β-bisabolene	495-61-4	nd	nd	nd	nd	0.77	nd	nd	nd	nd	nd
52	18.83	di-epi-α-cedrene	50894-66-1	nd	nd	nd	nd	nd	nd	nd	nd	nd	1.91
53	19.08	β-cedrene	546-28-1	nd	nd	nd	nd	0.36	nd	nd	nd	nd	nd
54	19.24	*(E)*-γ-bisabolene	53585-13-0	nd	nd	nd	nd	1.02	nd	nd	nd	nd	nd
55	19.71	widdrenal	470-41-7	nd	nd	nd	nd	nd	nd	nd	nd	nd	1.09
56	19.82	E-nerolidol	40716-66-3	nd	nd	nd	nd	nd	1.51	nd	nd	nd	nd
57	20.22	1h-cycloprop[e]azulen-7-ol	6750-60-3	nd	nd	nd	6.01	nd	nd	nd	nd	nd	nd
58	20.35	caryophyllene oxide	1139-30-6	nd	nd	nd	2.23	nd	nd	2.47	0.47	2.74	nd
59	20.57	guaiol	489-86-1	nd	nd	nd	nd	nd	nd	nd	0.12	nd	nd
60	20.70	epicurzerenone	20085-85-2	nd	nd	nd	nd	nd	nd	1.91	nd	nd	nd
61	20.84	germacrone	6902-91-6	nd	nd	nd	nd	nd	nd	nd	nd	nd	100.00
62	20.85	humulene epoxide ii	19888-34-7	nd	nd	nd	nd	nd	nd	nd	0.10	nd	nd
63	20.94	(1r,7s,e)-7-isopropyl-4,10-dimethylenecyclodec-5-enol	81968-62-9	nd	nd	nd	nd	nd	nd	nd	nd	nd	5.44
64	21.61	β-eudesmol	473-15-4	nd	nd	nd	nd	nd	1.21	nd	nd	nd	nd
65	22.85	β-spathulenol	77171-55-2	nd	nd	nd	2.42	nd	nd	nd	nd	nd	nd
66	22.90	(2r,3r,4ar,5s,8as)-2-hydroxy-4a,5-dimethyl-3-(prop-1-en-2-yl)-2,3,4,4a,5,6-hexahydronaphthalen-1(8ah)-one	5090-89-1	nd	nd	nd	nd	nd	nd	nd	nd	nd	1.66
67	23.32	xanthorrhizol	30199-26-9	nd	nd	nd	nd	nd	nd	nd	nd	nd	2.29
68	24.34	ambrial	3243-36-5	0.45	nd	4.56	7.19	nd	nd	0.29	0.59	0.43	nd
69	24.90	hexahydrofarnesyl acetone	502-69-2	nd	nd	1.25	nd	nd	nd	0.26	1.00	nd	nd

70	24.92	curcumenone	100347-96-4	nd	nd	nd	nd	nd	nd	nd	nd	nd	0.40
71	25.21	10,10-dimethyl-2,6-dimethylenebicyclo[7.2.0]undecan-5β-ol	19431-80-2	nd	nd	nd	nd	nd	nd	nd	nd	nd	1.11
72	25.35	dibutyl phthalate	84-74-2	0.31	nd	nd	nd	0.69	nd	nd	nd	nd	nd
73	25.35	2,4,7,14-tetramethyl-4-vinyl-tricyclo[5.4.3.0(1,8)]tetradecan-6-ol	1000193-31-2	nd	nd	nd	nd	nd	nd	0.33	nd	0.49	nd
74	26.18	farnesyl acetone	1117-52-8	nd	nd	2.35	nd	nd	nd	nd	nd	nd	nd
75	26.64	palmitoleic acid	373-49-9	nd	nd	nd	4.99	nd	nd	nd	nd	nd	nd
76	26.84	n-hexadecanoic acid	57-10-3	0.92	8.90	3.32	24.78	2.13	2.27	5.62	6.03	5.40	2.10
77	28.23	5-hydroxy-7-methoxyflavone	520-28-5	nd	nd	nd	nd	1.12	nd	nd	nd	nd	nd
78	29.03	vitamin e	59-02-9	nd	nd	nd	nd	nd	2.02	nd	nd	nd	nd
79	29.13	(1r,4ar,4bs,7s,10ar)-1,4a,7-trimethyl-7-vinyl-1,2,3,4,4a,4b,5,6,7,8,10,10a-dodecahydrophenanthrene-1-carbaldehyde	1686-63-1	nd	nd	nd	nd	2.28	nd	nd	nd	nd	nd
80	29.21	phytol	150-86-7	nd	nd	nd	nd	nd	2.08	0.98	1.08	0.89	6.07
81	29.45	4-methoxy-6-phenethyl-2h-pyran-2-one	3155-51-9	2.70	100.00	3.38	nd	nd	100.00	nd	nd	nd	nd
82	29.49	9,12-octadecadienoic acid (z,z)-	60-33-3	0.47	2.67	nd	5.81	2.04	nd	1.38	4.33	4.11	0
83	29.56	coronarin e	117591-81-8	nd	nd	2.49	nd	nd	nd	nd	nd	nd	nd
84	29.58	oleic acid	112-80-1	nd	nd	nd	6.01	nd	nd	nd	nd	nd	nd
85	29.90	octadecanoic acid	57-11-4	nd	nd	nd	nd	nd	nd	0.24	nd	nd	nd
86	30.18	1-heptatriacotanol	105794-58-9	nd	nd	nd	nd	0.36	nd	nd	1.61	nd	nd
87	32.63	5,6-dehydrokavain	15345-89-8	nd	19.34	nd	nd	nd	1.89	nd	nd	nd	nd
88	32.97	*(E)*-labda-8(17),12-diene-15,16-dial	104263-85-6	11.45	nd	94.17	105.61	nd	nd	14.05	9.22	10.14	nd
89	34.21	7-methyl-z-tetradecen-1-ol acetate	1000130-99-6	nd	nd	nd	nd	nd	nd	nd	nd	1.61	nd
90	34.31	octacosane	630-02-4	nd	9.13	nd	nd	nd	nd	nd	nd	nd	nd

Relative content = peak area of substance/peak area of cyclohexanone ×10

Note: nd- stand for no detection
